# Variant rs2237892 of *KCNQ1* Is Potentially Associated with Hypertension and Macrovascular Complications in Type 2 Diabetes Mellitus in A Chinese Han Population

**DOI:** 10.1016/j.gpb.2015.05.004

**Published:** 2015-12-08

**Authors:** Wanlin Zhang, Hailing Wang, Xiaomin Guan, Qing Niu, Wei Li

**Affiliations:** 1 Zhejiang Key Laboratory of Medical Genetics, Wenzhou Medical University, Wenzhou 325035, China; 2 School of Laboratory Medicine and Life Science, Wenzhou Medical University, Wenzhou 325025, China

**Keywords:** *KCNQ1*, Type 2 diabetes mellitus, High-resolution melting analysis, Hypertension, Macrovascular disease, Single nucleotide polymorphism

## Abstract

***KCNQ1*** has been identified as a susceptibility gene of **type 2 diabetes mellitus** (T2DM) in Asian populations through genome-wide association studies. However, studies on the association between gene polymorphism of ***KCNQ1*** and T2DM complications remain unclear. To further analyze the association between different alleles at the **single nucleotide polymorphism** (SNP) rs2237892 within ***KCNQ1*** and TD2M and its complications, we conducted a case-control study in a Chinese Han population. The C allele of rs2237892 variant contributed to susceptibility to T2DM (odds ratio [OR], 1.45; 95% confidence interval [CI], 1.20–1.75). Genotypes CT (OR, 1.97; 95% CI, 1.24–3.15) and CC (OR, 2.49; 95% CI, 1.57–3.95) were associated with an increased risk of T2DM. Multivariate regression analysis was performed with adjustment of age, gender, and body mass index. We found that systolic blood pressure (*P* = 0.015), prevalence of **hypertension** (*P* = 0.037), and risk of **macrovascular disease** (OR, 2.10; CI, 1.00–4.45) were significantly higher in subjects with the CC genotype than in the combined population with genotype either CT or TT. Therefore, our data support that ***KCNQ1*** is associated with an increased risk for T2DM and might contribute to the higher incidence of **hypertension** and macrovascular complications in patients with T2DM carrying the risk allele C though it needs further to be confirmed in a larger population.

## Introduction

Type 2 diabetes mellitus (T2DM) is becoming increasingly prevalent throughout the world. The number of people living with diabetes is expected to increase from 387 million in 2014 to 592 million by 2035 according to the 6th Edition of the International Diabetes Federation’s (IDF) Diabetes Atlas [1]. In China alone, there are more than 113.9 million adults with diabetes and another 493.4 million with prediabetes up to 2013 [2]. Although lifestyle changes, an increasing prevalence of obesity, and an increasingly aging population are important drivers of this epidemic, genetic factors also play a major role in T2DM susceptibility [3]. The extensive application of genome-wide association studies (GWAS) in the identification of common genetic variants has greatly contributed to the discovery of diabetes susceptibility genes. To date, at least 40 genetic loci have been convincingly associated with T2DM, including *KCNQ1*, *CDKAL1*, *TCF7L2*, *HMG20A*, *HNF4A*, *HNF1B*, and *DUSP9* [4], [5], [6].

The potassium channel, voltage gated KQT-like subfamily Q, member 1 encoded by *KCNQ1* is essential for the repolarization phase of the cardiac action potential. KCNQ1 protein can form heteromultimers with two other potassium channel proteins, KCNE1 and KCNE3. It is well known that *KCNQ1* gene mutations could result in hereditary long QT syndrome 1, Jervell and Lange-Nielsen syndrome, and familial atrial fibrillation [7]. *KCNQ1* is also expressed in insulin-producing cells. Inhibition of KCNQ1 channel activity by the selective inhibitor chromanol 293B significantly increases insulin secretion in INS-1 cells [8], whereas *KCNQ1* overexpression in MIN6 cells results in markedly impaired insulin secretion by glucose, pyruvate, or tolbutamide [9].

Multiple genetic variants have been identified in *KCNQ1*, including three main single nucleotide polymorphisms (SNPs) located in the intron 15 of *KCNQ1*, *i.e.*, rs2237892, rs2237895, and rs2237897. Several GWAS analyses showed that these variants are associated with T2DM and impaired insulin secretion in different populations including Asians, Europeans, and American Indians [10], [11], [12]. In addition, the SNPs of *KCNQ1* such as rs2074196, rs2237892, and rs2237895, were demonstrated to be associated with the risk of gestational diabetes mellitus in Koreans [13], and rs2283228 might contribute to the susceptibility of East Asians (Japanese and Singaporeans) to diabetic nephropathy [14]. These findings indicate that *KCNQ1* variants are clearly associated with a range of pathological conditions. However, whether *KCNQ1* variants are associated with the diseases other than long QT syndrome and diabetes need to be further examined.

As mentioned above, SNP rs2237892 has been reported to be associated with T2DM in the population of Asians, Europeans, and American Indians. It has also been investigated in several studies in the Chinese population [15], [16], however, these studies showed conflicting results. In this study, we chose the SNP rs2237892, the most common SNP of *KCNQ1*, to explore its association with not only T2DM but also its complications in a Chinese Han population from Wenzhou, Zhejiang province, for which there was no related report yet. Based on our data, we confirmed that rs2237892 was associated with an increased risk of T2DM in the population. We also found that compared with genotypes TT and CT, genotype CC was associated with a tendency of increasing incidence of hypertension and macrovascular complications in patients with T2DM.

## Results

### Patients’ clinical characteristics

A total of 530 subjects diagnosed with T2DM and 452 non-diabetic control subjects were recruited in this study. Compared to controls, T2DM patients had significantly higher blood pressure and higher levels of fasting blood glucose (FBG), triglycerides, and total cholesterol, but significantly lower level of high-density lipoprotein (HDL) (*P* < 0.001 for each variable) (Table 1). These data indicated the typical clinical manifestations of T2DM and metabolic syndrome.

### Genotyping rs2237892 variants in T2DM patients

**Figure 1:**
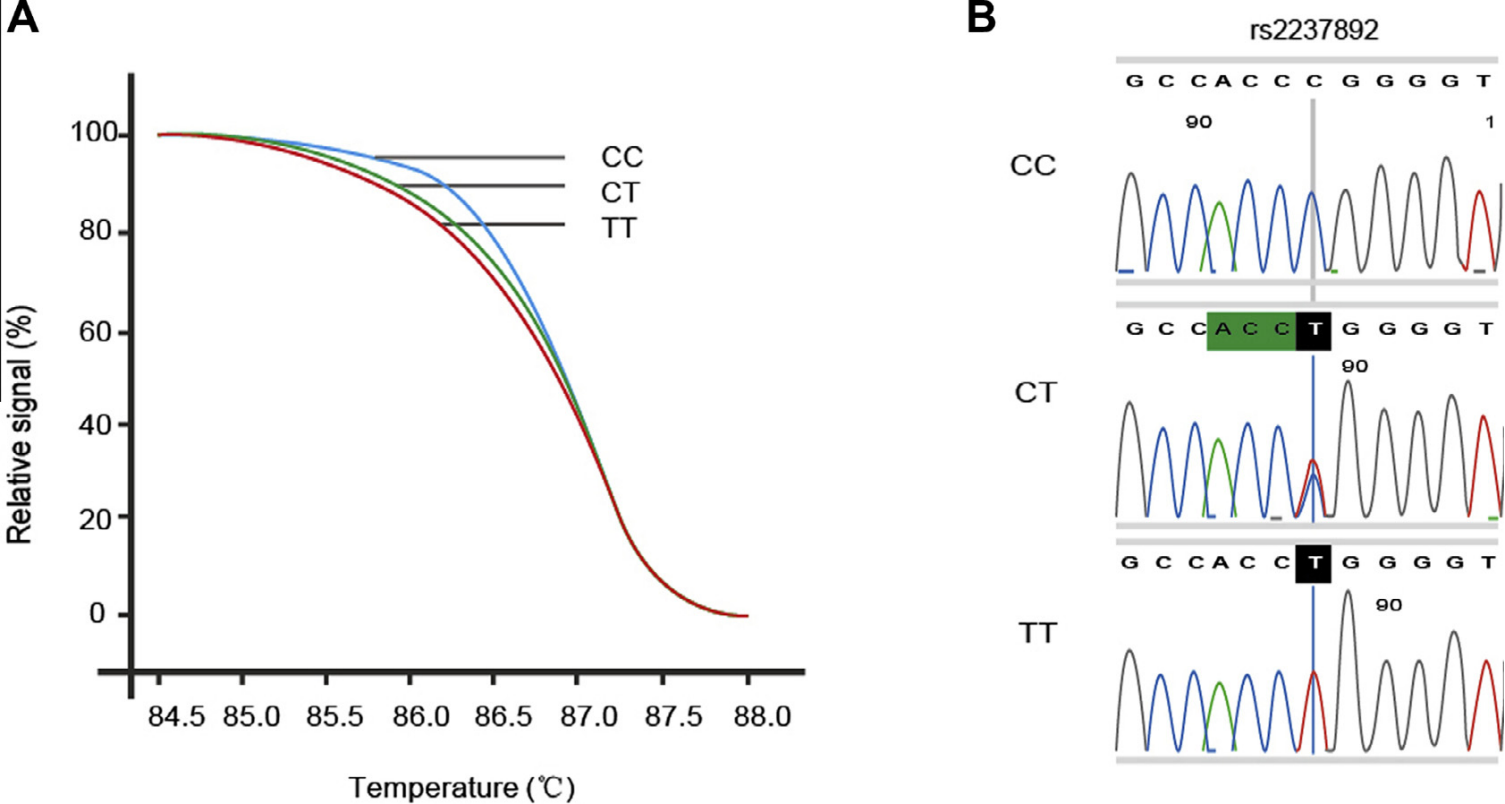
**Genotyping of rs2237892 variant in *KCNQ1*** **A.** Genotyping of rs2237892 was performed using HRM analysis. Relative fluorescence intensity was plotted for different genotypes at indicated temperature. **B.** HRM genotyping results were validated by DNA sequencing. Genotypes were identified as CC, CT, and TT. HRM, high-resolution melting

Genotypes of the SNP rs2237892 were determined according to the results of high-resolution melting (HRM) and DNA sequencing (Figure 1). The genotype distribution is summarized in Table 2. The genotype distribution of the variant was in accordance with Hardy–Weinberg equilibrium among the subjects (*P* > 0.05). The distribution of the genotypes between T2DM patients and controls was significantly different (*P* < 0.001). When the TT genotype was set as the reference, both the CT and CC genotypes were associated with an increased risk of T2DM (OR, 1.97; 95% CI, 1.24–3.15 for CT; and OR, 2.49; 95% CI, 1.57–3.95 for CC, respectively, Table 2). The allelic frequencies of the variant also differed significantly between the two cohorts (*P* < 0.001). With the T allele of rs2237892 set as the reference, the C allele was associated with an increased risk of T2DM (*P* < 0.001, OR, 1.45; 95% CI, 1.20–1.75). Thus, our findings are consistent with previous studies showing that the risk allele of the rs2237892 variant in *KCNQ1* might contribute to susceptibility to T2DM [11], [12], [13].

### Association between genotypes and clinical characteristics of T2DM patients

We then investigated the association between the three genotypes and the clinical characteristics of the T2DM subjects. Using the CC/(CT + TT) model, we found that systolic blood pressure was significantly higher in patients with genotype CC than in the combined group with genotype CT or TT (*P* = 0.03), even with multivariate logistic regression analysis adjusted for age, gender, and BMI (*P* = 0.015) (Table 3). None of the other variables differed significantly between the genotypes.

We also classified patients based on the presence of primary hypertension (systolic blood pressure ⩾140 mmHg or diastolic blood pressure ⩾90 mmHg). Adjusted multivariate logistic regression analysis indicated that high systolic blood pressure, but not high diastolic blood pressure, was significantly more prevalent in patients with the CC genotype than in those with the CT/TT genotypes (*P* = 0.037) (Table 4). These data suggest that the CC genotype might contribute to a higher occurrence of hypertension among subjects with T2DM.

### Association between genotypes and diabetic complications in T2DM patients

To assess the association of the three genotypes and the two alleles of the rs2237892 variants in the aggravation of diabetic complications, we divided the T2DM patients into four groups: macrovascular disease, diabetic retinopathy, diabetic nephropathy, and diabetic neuropathy. The frequency of both macrovascular disease and diabetic retinopathy increased in the order from genotypes TT to CT to CC, suggesting that the risk allele may be associated with susceptibility to both macrovascular disease (OR, 1.29; 95% CI, 0.99–1.69; *P* = 0.06) and diabetic retinopathy (OR, 1.28; 95% CI, 0.98–1.67; *P* = 0.07), although the associations were not significant (Table 5). We thus performed the multivariate logistic regression analysis for the genotypes after adjusted by age, gender, and BMI. Interestingly, we found that, compared to the TT genotype, the CC genotype showed the tendency to increase the risk of macrovascular disease in T2DM patients (OR, 2.10; 95% CI, 1.00–4.45; *P* = 0.05).

## Discussion

*KCNQ1* mutations are associated with cardiac diseases such as hereditary long QT syndrome and familial atrial fibrillation [7]. As a primary potassium channel subunit, KCNQ1 is expressed in other tissues as well, including the brain, adipose tissue, and pancreas [17], [18], [19]. In 2008, it was demonstrated that three variants of rs2283228, rs2237895, and rs2237895 within *KCNQ1* were strongly associated with an increased risk of T2DM in East Asian and European populations [11], and rs2237892 was associated with type 2 diabetes in two independent Japanese populations, as well as Korean, Chinese, and European ancestry [20]. For the Chinese population, it has been confirmed in several independent studies that these variants of *KCNQ1* could confer susceptibility to T2DM, however, various studies showed conflicting results in terms of the variants involved [15], [16], [21], and the association has not been verified in the population of Wenzhou.

In the present study, we examined the contribution of the variant rs2237892 to the risk of T2DM and its complications in the Chinese Han population from Wenzhou of Zhejiang province, which is in southeast China. We also found a significant association with T2DM in that the C allele conferred an increased risk of the disease, which was consistent with previous reports [21], [22], [23].

*KCNQ1* confers a risk for T2DM by impairing β-cell function [24]. The variants (rs2074196, rs2237892, rs2237895, and rs2237897) are significantly associated with impaired FBG [25] and reduced insulin release following an oral glucose load [26]. Other studies have indicated that *KCNQ1* is associated with obesity [16] and triglyceride levels [27] in Chinese Han populations. Polymorphisms in the *KCNQ1* gene were reportedly related with the therapeutic efficacy of repaglinide in treating Chinese patients with T2DM [28]. However, in our study, we failed to replicate the significant differences in BMI, FBG, and lipid levels among T2DM subjects with different genotypes, possibly as a result of the low power of our study to detect quantitative traits, probably suggesting that *KCNQ1* is not a major factor associated with these traits. Nevertheless, using multivariate logistic regression analysis with adjustment for age, gender, and BMI, we discovered that both systolic blood pressure and the prevalence of hypertension were significantly higher in T2DM patients with the CC genotype than in those with the CT/TT genotypes. The contemporary incidence of hypertension and diabetes was distinct across different ethnic, racial, and social groups. Hypertension occurred in nearly 70% of diabetic patients and was approximately twice as common in individuals with diabetes than in those without diabetes [29]. It has been documented that Kv7 channels encoded by *KCNQ* gene family contributed to vasoconstriction and hypertension [30], which was in concert with the discovery that both C3H/HeJCrl-*Kcnq1*^vtg-2J^/J mice [31] and WTC-*dfk* rats [32] with a *KCNQ1* mutation exhibited significantly higher blood pressure. Taken together, these findings suggest that *KCNQ1* mediates the development of hypertension in patients with T2DM.

Hypertension in patients with diabetes greatly increases the risk of vascular complications in this population. Moreover, both hypertension and diabetes predispose an individual to chronic kidney disease [33]. Therefore, the increased risk of hypertension observed for T2DM patients with the CC genotype suggests a role for the risk allele C at rs2237892 in the development of diabetic complications. In our study, the prevalence of macrovascular disease increased in T2DM patients from the TT to the CT and finally the CC genotypes. Multivariate regression analysis after adjusted by age, gender, and BMI demonstrated that the T2DM patients with the CC genotype were twice as likely to have macrovascular complications as those with the TT genotype. Diabetic retinopathy and diabetic nephropathy also tended to be more prevalent with a genotype of CC than of TT.

In conclusion, the association of *KCNQ1* variant rs2237892 susceptibility to T2DM was replicated in a Chinese Han population. Moreover, although it needs to be further confirmed in a larger population, we discovered that the genotype CC of rs2237892 tended to be associated with the increased risk of hypertension and macrovascular complications in T2DM patients, suggesting a potential biomarker predicting the development of complications that could be identified at an early stage of T2DM.

## Materials and methods

### Subjects and samples

The diagnosis of T2DM was made in accordance with the World Health Organization (WHO) 1999 criteria [34] and the China Guideline for Type 2 Diabetes 2010 (Chinese Diabetes Society) [35]. Known subtypes of diabetes such as type 1 diabetes and secondary diabetes were excluded. All patients were admitted in hospital without previous treatment. Control subjects were individuals with a FBG level <6.1 mM and normal glucose tolerance, without family history of diabetes mellitus. Diabetic complications were diagnosed by diabetologists according to the clinical manifestations and a related auxiliary examination.

All participants were of Chinese Han origin, non-sanguineous, and recruited between May 2009 and October 2010 from the First Affiliated Hospital of Wenzhou Medical University, Wenzhou, China. Written informed consent was obtained from the subjects prior to participation. Clinical evaluations were approved by the Ethics Committee of the Wenzhou Medical University Institutional Review Board.

Height, body weight, and blood pressure were measured for all subjects. Blood samples were drawn for biochemical measurements: FBG, total cholesterol, triglyceride, HDL. Genomic DNA was extracted from peripheral whole blood using Takara blood DNA isolation kits (Takara, Dalian, China) and quantified using a NanoDrop ND-2000 spectrophotometer (Thermo Fisher Scientific, Waltham, MA).

### Genotyping using HRM

Genotyping was performed using a LightCycler 480 System II (Roche Diagnostics, Penzberg, Germany). A 116-bp fragment spanning the rs2237892 loci was amplified for subsequent HRM analysis with primers 5′-AGAGGAAGAGCAAGGGTAGG-3′ (forward) and 5′-GGTGTAAGGCATCTGGTGG-3′ (reverse), designed against GenBank sequence with accession No. NC_000011.10. PCR amplification was performed as instructed by the manufacturer. The amplification products were heated to 95 °C for 1 min and then cooled to 40 °C for 1 min. HRM was subsequently performed over a range of 65–95 °C, increasing at 1 °C/s with 25 acquisitions per 1 °C step. HRM curves were generated using the LightCycler 480 gene scanning software module with manual settings for sensitivity to 0.30, a temperature shift to threshold 4, a pre-melt normalization range of 70–78 °C, and a post-melt normalization range of 85–90 °C. The melting curves were normalized for direct comparison among samples [36].

### Genotyping using DNA sequencing

To confirm the HRM results, samples were randomly selected for DNA sequencing. A 500-bp fragment spanning the rs2237892 locus was amplified for DNA sequencing using the primers designed against GenBank sequence NC_000011.10, 5′-GTGCATCCTAAGGTGGTTC-3′ (forward) and 5′-CCTAATCCTGTAGGGCAGA-3′ (reverse) following standard protocol for PCR amplification. The PCR products were purified using a QIAquick PCR purification kit (Qiagen, Valencia, CA) and subsequently analyzed by direct sequencing with an ABI 3730XL automated DNA sequencer in BGI (Beijing, China).

### Statistical analysis

Quantitative clinical data (age, BMI, blood pressure, FBG, total cholesterol, triglyceride, and HDL) were presented as mean ± standard deviations and compared using a one-way analysis of variance. To correct for non-normally distributed data, concentrations of FBG, total cholesterol, triglyceride, and HDL were log-transformed. Gender, which is presented as binary data (male/female), was compared using the Chi-square test.

Genotype distribution and allelic frequencies are presented as number (%) and were analyzed using a Chi-square test or multivariate logistic regression analysis. Multivariate logistic-regression analysis was performed to adjust for risk factors, with T2DM as a dependent variable and independent variables including age, sex, and BMI. Results are presented as OR and 95% CI. All values of *P* < 0.05 were considered significant. The statistical analyses were performed with SPSS version 17.0 software (SPSS, Chicago, IL, USA).

## Authors’ contributions

WL and WZ conceived and designed the study. HW, MG, and QN performed the experiment and analyzed the data. WL and WZ wrote and revised the paper. All authors read and approved the final manuscript.

## Competing interests

The authors have declared that no competing interests exist.

## Figures and Tables

**Table 1:** Clinical characteristics of Chinese Han T2DM patients and controls

**Parameter**	**T2DM (n = 530)**	**Control (n = 452)**	***P* value**
Age (years)	60.95 ± 12.62	58.83 ± 11.40	0.019
Gender (male/female)	281/249	230/222	0.505
Body mass index (kg/m^2^)	24.24 ± 3.39	22.32 ± 2.20	<0.001

Blood pressure (mmHg)			
Systolic blood pressure	142.81 ± 24.7^#^	119.11 ± 11.51	<0.001
Diastolic blood pressure	80.23 ± 11.64^#^	74.65 ± 8.25	<0.001
Fasting blood glucose (mM)	8.89 ± 3.98	4.96 ± 0.64	<0.001
Total cholesterol (mM)	4.50 ± 1.18	4.81 ± 0.76	<0.001
Triglyceride (mM)	1.64 ± 1.27	1.16 ± 0.54	<0.001
High-density lipoprotein (mM)	1.18 ± 0.38	1.41 ± 0.31	<0.001

*Note:* Values are presented as mean ± SD except the gender. *P* values were determined using a one-way analysis of variance except Chi-square analysis for the gender. ^#^ Blood pressure data were missing for one T2DM patient. T2DM, type 2 diabetes mellitus.

**Table 2:** Genotype distribution and allele frequency among Chinese Han T2DM patients and controls

**Genotype or allele**	**Genotype frequency, number (%)**		***P* value**	**Unadjusted OR** **(95% CI)**	**Adjusted OR** **(95% CI)**
**Diabetes (n = 530)**	**Control (n = 452)**
Genotype					
TT	39 (7.36)	66 (14.60)	<0.001	1.00	1.00
CT	217 (40.94)	192 (42.48)		1.91 (1.23–2.97)	1.97 (1.24–3.15)
CC	274 (51.70)	194 (42.92)		2.39 (1.55–3.70)	2.49 (1.57–3.95)

Allele					
T	295 (27.83)	324 (35.84)	<0.001		
C	765 (72.17)	580 (64.16)		1.45 (1.20–1.75)	

*Note: P* values were determined using Chi-square test. OR was calculated using multivariate logistic regression analyses without and with adjustment for gender, age, and body mass index. T2DM, type 2 diabetes mellitus; OR, odds ratio; CI, confidence interval.

**Table 3:** Clinical characteristics of Chinese Han T2DM patients by genotype

**Parameter**	**Genotype**			***P* value**
**TT (n = 39)**	**CT (n = 217)**	**CC (n = 274)**
Age (years)	61.62 ± 11.91	60.55 ± 13.27	61.17 ± 12.21	0.675
Gender (male/female)	21/18	110/107	150/124	0.410
Body mass index (kg/m^2^)	24.43 ± 3.63	24.24 ± 3.58	24.21 ± 3.20	0.846

Blood pressure (mm Hg)				
Systolic blood pressure	139.38 ± 21.34	140.57 ± 24.33	145.06 ± 25.36	0.030 0.015^#^
Diastolic blood pressure	80.10 ± 11.53	79.13 ± 11.93	81.13 ± 11.40	0.067
Fasting blood glucose (mM)	9.97 ± 6.44	8.51 ± 3.34	9.03 ± 3.98	0.357
Total cholesterol (mM)	4.40 ± 1.04	4.41 ± 1.21	4.60 ± 1.18	0.070
Triglyceride (mM)	1.61 ± 1.34	1.72 ± 1.44	1.59 ± 1.10	0.527
High-density lipoprotein (mM)	1.17 ± 0.33	1.18 ± 0.37	1.18 ± 0.40	0.791

*Note:* Values are presented as mean ± SD except for the gender. *P* values were determined using a one-way analysis of variance except Chi-square analysis for the gender. *P* value with multivariate logistic regression analysis adjusted for age, gender, and body mass index is indicated by ^#^. Comparison between CC genotype *vs.* CT + TT genotypes was performed using recessive model. The blood pressure data were missing for one patient with CC genotype (*n* = 273). T2DM, type 2 diabetes mellitus.

**Table 4:** Association of genotypes with risk of hypertension in Chinese Han T2DM patients

**Blood pressure**	**Genotype, number (%)**			***P* value**
**TT (n = 39)**	**CT (n = 217)**	**CC (n = 273)**
Systolic				
<140 mmHg	18 (46.15)	103 (47.47)	105 (38.46)	0.037
⩾140 mmHg	21 (53.85)	114 (52.53)	168 (61.54)	

Diastolic				
<90 mmHg	30 (76.92)	174 (80.18)	209 (75.56)	0.340
⩾90 mmHg	9 (23.08)	43 (19.82)	64 (23.44)	

*Note: P* values were determined using Chi-square analysis. Multivariate logistic regression analysis was performed after adjusted for age, gender, and body mass index. The comparison between CC genotype *vs.* CT + TT genotypes was performed using recessive model.

**Table 5:** Association of genotypes with risk of diabetic complications in Chinese Han T2DM patients

**Complication**	**Genotype, number (%)**			***P* value**			**OR (95% CI)^a^**	**OR (95% CI)^c^**
**TT**	**CT**	**CC**	***P*^a^**	***P*^b^**	***P*^c^**
Macrovascular disease	15 (38.46)	105 (48.39)	147 (53.65)	0.06	0.16	0.05	1.29 (0.99–1.69)	2.10 (1.00–4.45)
Diabetic retinopathy	17 (43.59)	101 (46.54)	148 (54.01)	0.07	0.18	0.21	1.28 (0.98–1.67)	1.55 (0.78–3.08)
Diabetic nephropathy	7 (17.95)	63 (29.03)	77 (28.10)	0.46	0.36	0.17	1.12 (0.83–1.52)	1.85 (0.77–4.43)
Diabetic neuropathy	24 (61.54)	108 (49.77)	149 (54.38)	0.96	0.32	0.40	1.01 (0.77–1.32)	0.74 (0.37–1.48)

*Note: P* values for allele frequency (^a^) and genotype distribution (^b^) were determined using Chi-square test. Multivariate logistic regression analysis was performed for CC genotype *vs.* TT genotype after adjusted for age, gender, and body mass index (^c^). T2DM, type 2 diabetes mellitus.
